# MORT: a powerful foundational library for computational biology and CADD

**DOI:** 10.1186/1758-2946-6-36

**Published:** 2014-06-27

**Authors:** Qian Zhang, Wei Zhang, Youyong Li, Junmei Wang, Jian Zhang, Tingjun Hou

**Affiliations:** 1 Institute of Functional Nano & Soft Materials (FUNSOM) and Collaborative Innovation Center of Suzhou Nano Science and Technology, Soochow University, Suzhou, Jiangsu 215123, China; 2 College of Pharmaceutical Sciences, Zhejiang University, Hangzhou, Zhejiang 310058, P. R. China; 3 BGP Research and Development Center at Houston, 10630 Haddington Dr, Houston, TX 77043, USA; 4 Department of Biochemistry, University of Texas Southwestern Medical Center, 5323 Harry Hines Blvd., Dallas, TX 75390, USA; 5 Department of Pathophysiology, School of Medicine, Shanghai Jiao-Tong University, Shanghai 200025, P. R. China

**Keywords:** Relational model, MORT, AMBER, Antechamber, Foundational library, CADD

## Abstract

**Background:**

A foundational library called MORT (Molecular Objects and Relevant Templates) for the development of new software packages and tools employed in computational biology and computer-aided drug design (CADD) is described here.

**Results:**

MORT contains several advantages compared with the other libraries. Firstly, MORT written in C++ natively supports the paradigm of object-oriented design, and thus it can be understood and extended easily. Secondly, MORT employs the relational model to represent a molecule, and it is more convenient and flexible than the traditional hierarchical model employed by many other libraries. Thirdly, a lot of functions have been included in this library, and a molecule can be manipulated easily at different levels. For example, it can parse a variety of popular molecular formats (MOL/SDF, MOL2, PDB/ENT, SMILES/SMARTS, etc.), create the topology and coordinate files for the simulations supported by AMBER, calculate the energy of a specific molecule based on the AMBER force fields, etc.

**Conclusions:**

We believe that MORT can be used as a foundational library for programmers to develop new programs and applications for computational biology and CADD. Source code of MORT is available at http://cadd.suda.edu.cn/MORT/index.htm.

## Background

Molecular modeling techniques have been widely used in the fields of chemistry, biology, drug design, and materials science for studying molecular systems ranging from small molecules to large biological molecules and even material assemblies. A lot of molecular simulation and visualization tools or packages have been developed [1-9]. Amber [6] is extensively used for the simulation of biomolecules, and XLEAP is its graphical user interface. XLEAP is written in C but trying to use the object-oriented programming (OOP) paradigm that is not natively supported by C. Therefore, the code of XLEAP is awkward and extremely hard to be extended. Consequently, a lot of functionalities that should have been incorporated into XLEAP were either implemented as separate programs, such as ADDLES2 for Locally Enhanced Sampling, and antechamber for the calculations of partial charges and the assignment of atom types, or merged into molecular dynamics (MD) calculation stage as extra points. The study we discuss here is trying to put this situation to an end by developing a new foundational library that is called Molecular Objects and Relevant Templates (MORT).

MORT is written in C++ that is a native OOP language, and a relational model and other well-designed patterns have been applied to this library, which makes it very flexible. Furthermore, many commands/functions supported by XLEAP and AmberTools have been merged into this library [6]. Based on MORT, it will be very easy for programmers or readers with interests to develop new applications for computational biology and CADD.

## Implementation

### Data structure and basic features of MORT

#### The relational model of MORT

An advantage of MORT is that it employs the relational model rather than the traditional hierarchical model to store all the information of a molecule. In the hierarchical model, a molecule owns some residues, and a residue owns some atoms. Therefore, a molecule does not own atoms directly. In the relational model, a molecule owns residues and atoms directly, while a residue does not own atoms, and they just have relations between each other.

The hierarchical model is used by XLEAP and some other software packages (for example, NAB [10]). But it has the following disadvantages. Firstly, the hierarchical model is inconvenient for iterating over the atoms in a molecule. In the hierarchical model, to iterate over atoms, users need to iterate over all residues first. Therefore, counting atom number is not a constant time operation but proportional to the number of residues. To overcome this problem, some kind of cache may be used to store the atom number or pointers to the atoms inside a molecule. This solves the efficiency problem but brings up a consistency problem, i.e. the cache needs to be updated when an atom is deleted or inserted. Secondly, the hierarchical model has problems to handle bonds, because in the hierarchical model, residues own atoms, and it is natural that bonds should be owned by residues too. A problem may always exist for inter-residue bonds, whose ownership would be a dilemma for the hierarchical model. Similar things happen to other components, such as angles, torsions and improper torsions. Thirdly, the hierarchical model is hard to be extended. For example, adding a new object strand is a very useful concept for a DNA system, because a DNA molecule has two strands and each strand has several residues. In the hierarchical model, we need to add an extra layer between molecule and residue, but in practice, all code that involves the iterations on atoms and residues needs to be changed to iterate on strands first, which in turn means massive changes to the existing code.

However, all these problems do not exist or can be easily fixed for the relational model employed in MORT. Firstly, the problem of iterating on atoms does not exist because a molecule owns atoms directly, and then users can perform the iteration on a molecule directly disregarding residues. Secondly, the relational model does not have any problem dealing with bonds, angles and torsions, because in the relational model a molecule directly owns bonds, angles (if any) and torsions (if any). Finally, compared with the hierarchical model, the relational model is extremely easy to be extended. Taking the strand as an example, to introduce a strand by employing the relational model, users do not need to change the old code. Strand can only appear in DNA-related program and all users need to do is to make sure that the relations between strand and residues have been created correctly.

**Figure 1:**
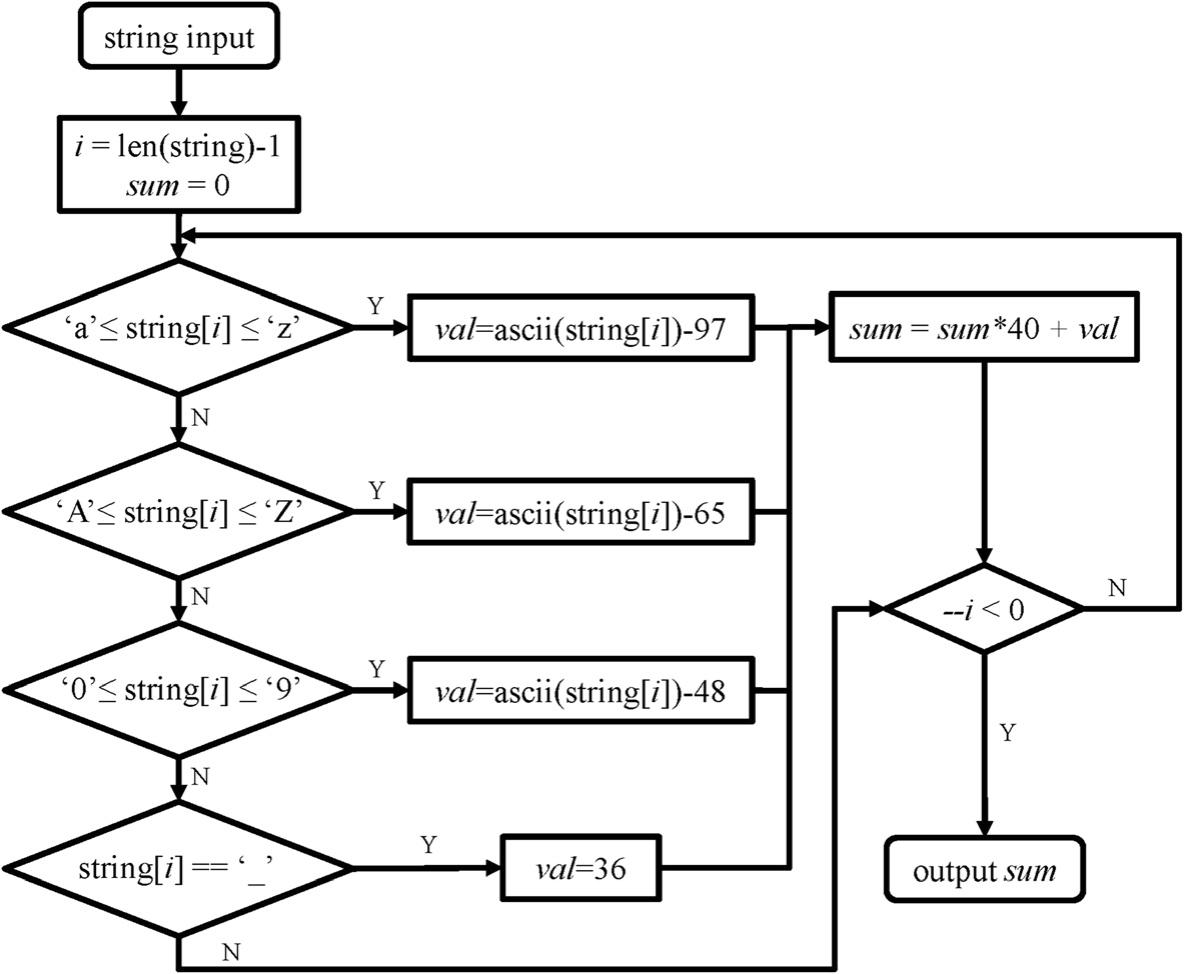
**The flowchart for the generation of a HASH value.** This function will iterate through each character of the input, and the returned value differs according to the type of the character: 1. an alphabetical character, its position comparing to ‘a’ (if it is lowercase) or ‘A’ (if it is uppercase) will be returned (the positions of ‘a’ and ‘A’ are set to 0); 2. a digit, the value of itself will be returned; 3. an underline, the value will be set to 36. If it does not belong to any shown above, the function will do nothing and continue to calculate the next character. At the end of each iteration, the value of one character will be added to the sum that is multiplied by 40 to get a new sum.

It has been mentioned that in the relational model, atoms, bonds and residues are owned directly by a molecule, so are angles, torsions and any other possible components such as strands and improper torsions. In MORT, these objects (atoms, bonds, etc.) are called molecular objects (referred as MOs). Currently, MORT supports eight types of MOs: atom, bond, residue, angle, torsion, out-of-plane stretch (also known as improper torsion), torsion-torsion interaction, and pi-torsion (the latter two are used exclusively by the AMOEBA force field). A four-character-long-code is assigned to each MO type, and they are *atom*, *bond*, *resd*, *angl*, *tors*, *oops*, *tor2* and *ptor*.The names of these object types are encoded into HASH values in order to save the time of comparing characters. Each HASH is 10 letters long and only composed of letters, digital characters and underlines. Some HASH values are predefined, and the others can be generated by using a function with a string as the input. The flowchart for the generation of a HASH value is illustrated in Figure 1.

#### Composition of a molecule

The basic idea of MORT is: a molecule (represented by class *molecule_t*) owns several MOs (atoms, bonds, etc.), and each MO has its own properties and there are relations between them. In order to save the properties and relations, two variables are created. The properties are stored in components (represented by *m_components*), and the relations are stored in adjacencies (represented by *m_adjacencys*). Both of *m_components* and *m_adjacencys* are the member variables of class *molecule_t*. A molecule usually has several components (i.e. an atom component, a bond component, and even a residue component in many cases) and has several adjacencies (i.e. atom-atom adjacency, atom-bond adjacency, bond-atom adjacency, atom-residue adjacency, and residue-atom adjacency). Descriptions of the important classes of variables are listed in Table 1.

**Table 1:** The descriptions of some important classes of variables

**Class**	**Description**
*molecule_t*	a class contains molecular objects and their relationships
*mcmpdata_t*	a class used to store the information of molecular objects
*mcmprela_t*	a class used to store the relationships between molecular objects
*database_t*	a class contains lots of molecules
*morf_t*	a base class of the molecular objects
*atom_t*	a class used to handle atoms
*bond_t*	a class used to handle bonds
*angl_t*	a class used to handle angles
*dihe_t*	a class used to handle dihedral angles
*resd_t*	a class used to handle residues

A component physically contains all the properties of a kind of molecular objects. For example, the atom component of a molecule contains all the properties of atoms. More specifically, the properties of the same type are stored sequentially in an array, the atomic numbers of all atoms are stored in an integer array, while the names of all atoms are stored in a string array. However, the properties whose type is numerical vector are handled differently, and they are not stored in an array of numerical vectors (which can be considered as a 2D array of floating numbers) but in a 1D array of floating numbers. The component is implemented in this way to achieve the best space efficiency.

An adjacency records the relations between two components. For each pair of connected molecular objects, there is a record in the adjacency. More specifically, it uses a 2D array to store connections.

**Figure 2:**
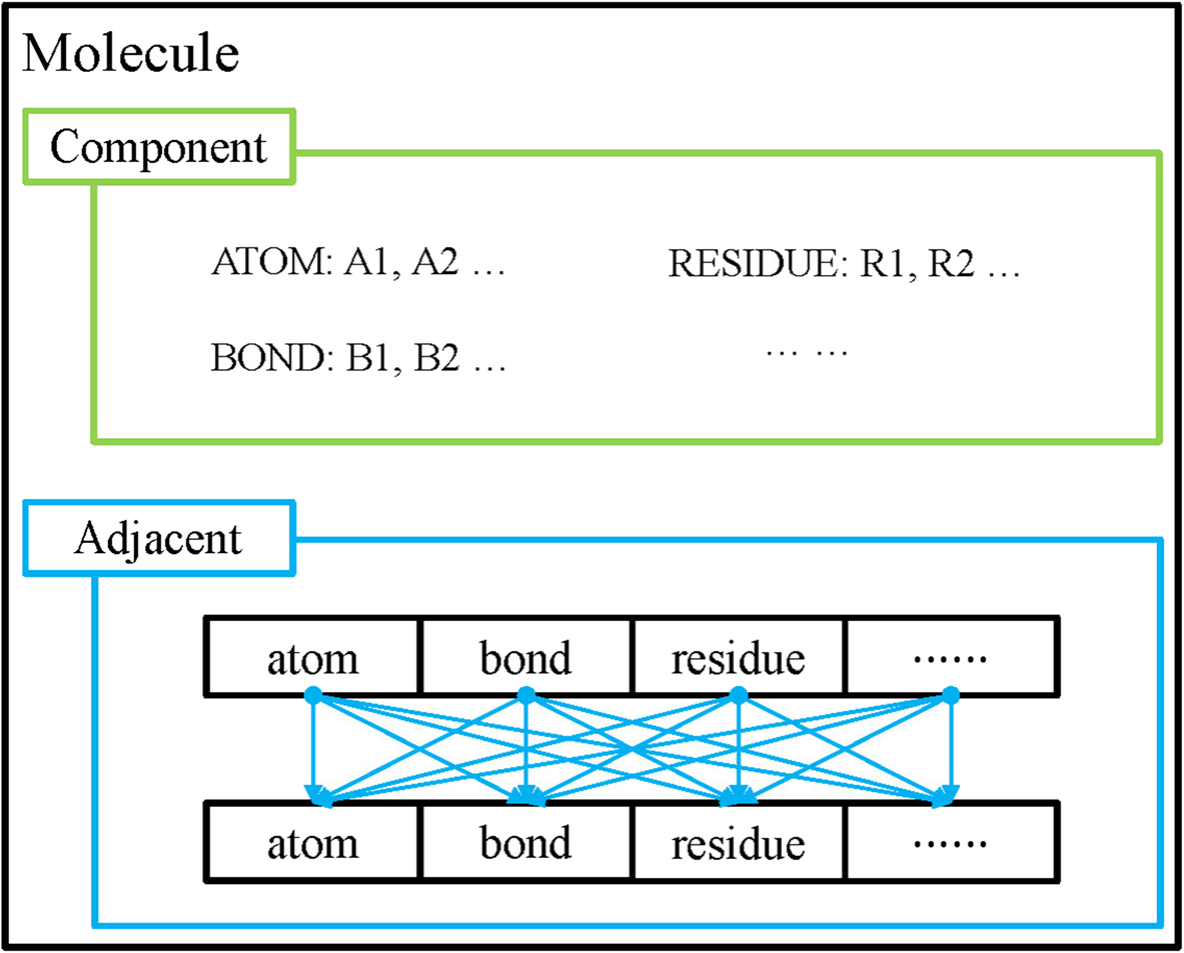
The composition of a molecule.

The composition of a molecule is shown in Figure 2. If users want to handle a MO, such as creation or deletion, they can operate with class *morf_t*, which is the base class for all MO classes. In brief, class *molecule_t* is designed for the storage of data, whereas class *morf_t* is created for the modification, creation or deletion of objects, and it does not contain any data information.

**Figure 3:**
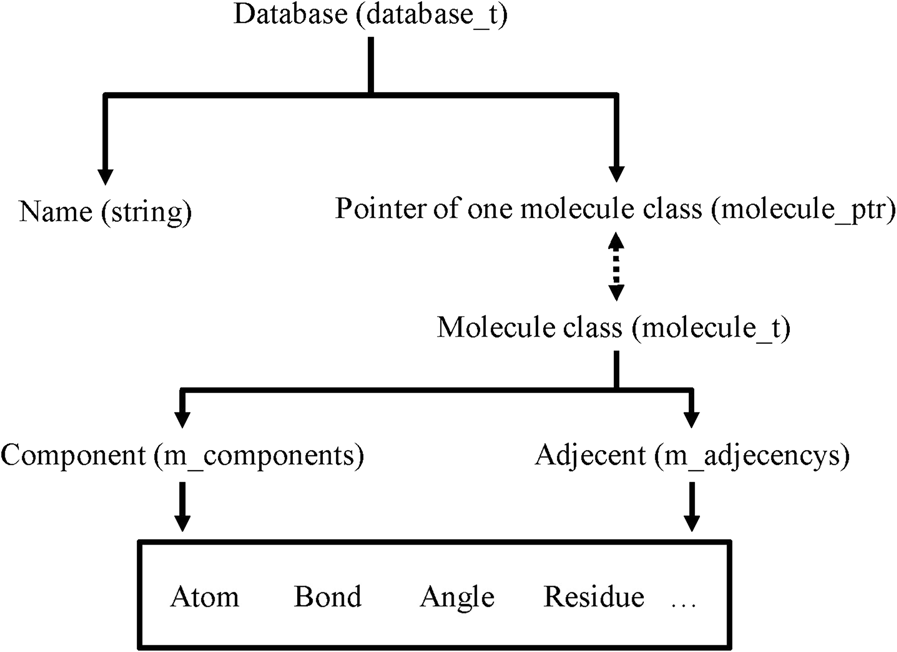
**The structure of class *database_t*.** Contents in the bracket are their corresponding classes.

Except for class *molecule_t*, another class (represented by class *database_t*) is created to store the information of a molecule: the molecule’s pointers and their corresponding names. Therefore, if a molecule needs to be modified, its corresponding pointers will be returned from the database by using the function *get_mol* with its name as the parameter. The structure of class *database_t* is shown in Figure 3. Classes *database_t* and *molecule_t* are both inherited from class *entity_t*, which is the base class for the storage of data.

#### Assessing properties of MOs

As has been mentioned before, class *morf_t* is created for the modification, creation or deletion of objects. In order to achieve the above goals, some functions are necessary to access the properties of MOs, and therefore several member functions have been designed as follows:

void set_x( const string& pname, const value_type& v );

value_type get_x( const string& pname ) const;

bool get_x( const string& pname, value_type& v );

The character ‘*x*’ in the names of these functions can be any of the following five characters: ‘*i*’ (for integer), ‘*d*’ (for double precision), ‘*s*’ (for string), ‘*v*’ (for numeric vector) and ‘*a*’ (for any other data type), while the *value_type* can be int, double, string, numvec and boost::any depending on the ‘*x*’. In order to accelerate the process, string can be replaced by HASH values, and these functions can be transformed into the following ones:

void set_x( const long long& pid, const value_type& v );

value_type get_x( const long long& pid ) const;

bool get_x( const long long& pid, value_type& v );

#### Iterating on MOs

Two methods can be used to iterate on the MOs of a molecule: MOITER (molecular object iterator, represented by class *iter_T*) or MORANGE (molecular object range, represented by class *range_T*).

MOITER is a random access iterator, and it has the following member functions:

morf_t& operator*();

morf_t const& operator*() const;

morf_t & operator- > ();

morf_t const& operator- > () const;

iter_t& operator++();

iter_t& operator--();

iter_t operator++(int);

iter_t operator--(int);

iter_t& operator + =(ptrdiff_t pdif);

iter_t& operator- = (ptrdiff_t pdif);

With all these member functions implemented, MOITER works just like a pointer to class *morf_t*. The following two member functions of class *molecule_t* return the starting and ending iterators of a certain type of MO:

iter_t xxxx_begin ();

iter_t xxxx_end();

Here, *xxxx* could be any of the eight 4 character ID of a molecular object type.

Another way to enumerate MOs is to use MORANGE (molecular object range, represented by class *range_T*). Class *range_T* has the following member functions:

morf_t operator[](int id) const;

morf_t at(int id) const;

MORANGE in a sense works just like an array of MOs. Class *molecule_t* has the functions *xxxxs()* that return the MORANGE of a certain type of MOs, while class *morf_t* has similar member functions *related_xxxxs()* that returns the MORANGE of related MOs.

### Basic functions in MORT

**Figure 4:**
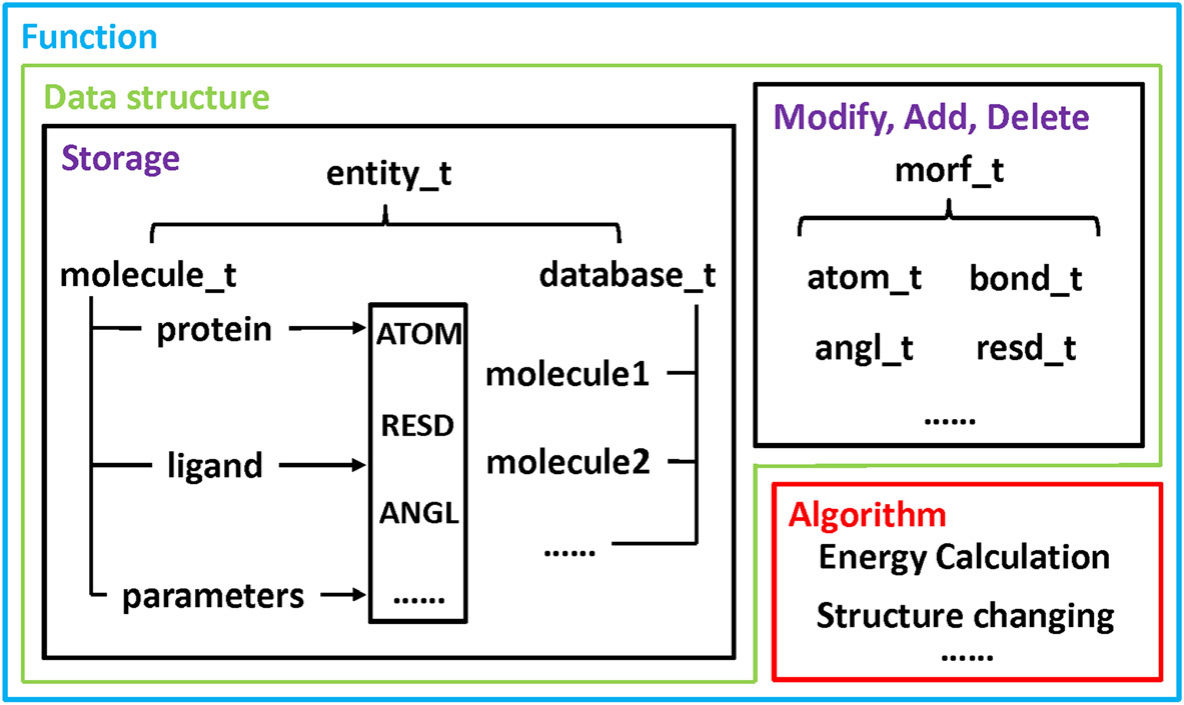
The composition of a function.

A lot of functions supported by Antechamber and XLEAP have been developed in MORT, and therefore based on MORT it is very easy for users to develop new applications for computational biology and CADD. As shown in Figure 4, a function in MORT is composed of data structure and algorithm. Algorithms are the operations that can be applied by the users to the target, and data structure is separated into two parts: one for information storage and the other for handling MOs. Proteins, ligands and parameters are stored in molecules (represented by class *molecule_t*), and are composed of MOs (atom, residue, etc.). Molecules are stored in database (represented by class *database_t*). Both of *molecule_t* and *database_t* are inherited from class *entity_t*. Class *morf_t* and its child classes are used to modify, add and delete MOs. The functions in MORT can be roughly divided into two categories: object-related and property-related.

#### Object-related functions

Functions in this category can handle the MOs defined in MORT. These functions have various usages, and they are distributed in different directories.

**Figure 5:**
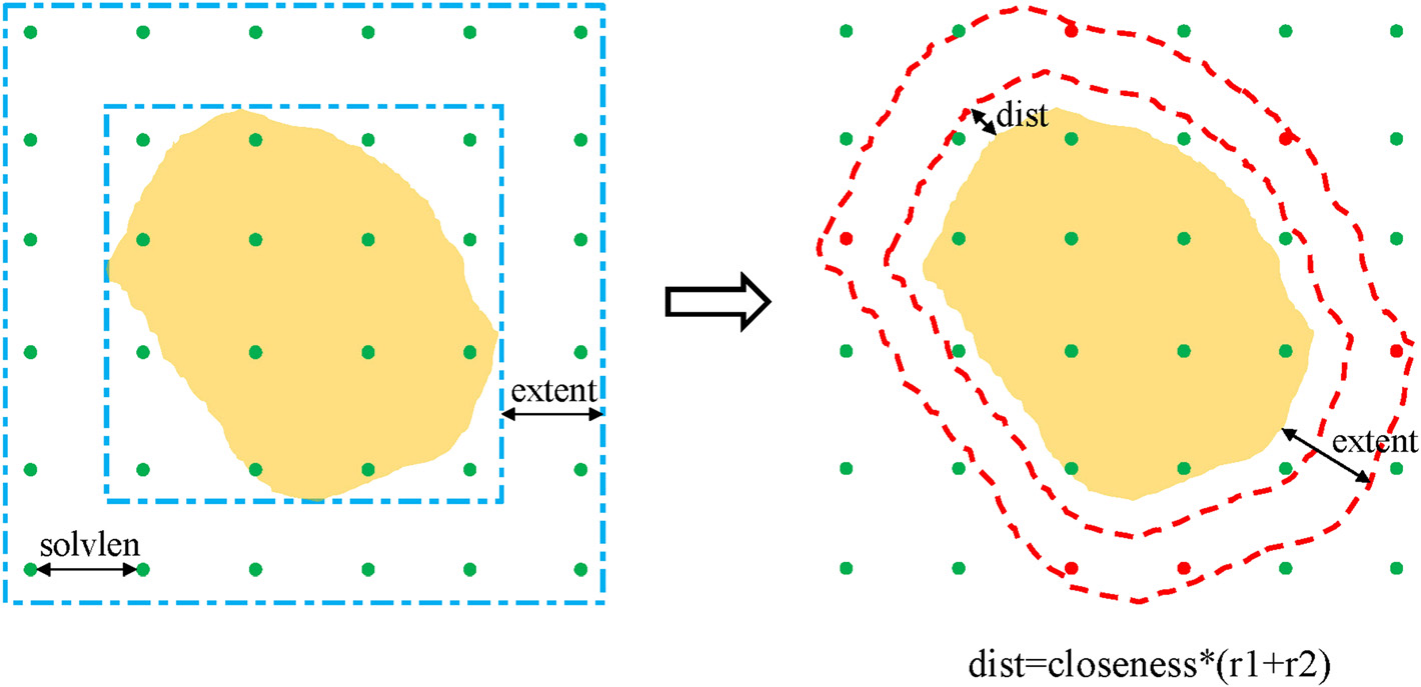
**A 2D schematic shows how *solvateshl *works.** *solvlen* equals to the size of one solvate, *r1*/*r2* equals to the van der Waals radius of the solute/solvent atom; *extent* and *closeness* are the parameters of this function, *closeness* represents the closeness between solute and solvent, and *extent* is used to determine the border of shell. The system will first be covered with a box, and then the box will be stretched to a big one with the length equal to *extent*. Lots of grids will be generated to represent the centroids of solvents with interval equal to *solvlen* in the box. For each atom in solvent, distance will be calculated between itself and the atoms in solute and the shortest one will be returned; if it is smaller than *dist or* longer than *extent*, the solvent that it belongs to will be excluded from the shell. After checking all the solvents, only a part of them can remain (marked as red), and the others (marked as green) will be neglected.

(1). **OBJFUN**: a lot of functions are defined in this directory, and they can be used to modify objects. For example, the function *fixbond* can be used to fix the bond order of a molecule based on several rules, such as hard rule, length rule and conjugation rule, as illustrated in our previous work [11]; the function *addHs* can be used to add the missing hydrogen atoms of a molecule (bond information is required); the function *create* can be used to create atoms, bonds, residues, angles, etc.; the functions *transform* and *translate* can be used to transform/move a molecule according to one matrix/vector; the function *rotate* can be used to rotate a molecule; the function *center* can be used to determine the geometrical center of a molecule or residue. A lot of other functions are not mentioned here, and the descriptions of the important functions can be found in Additional file 1. Apart from being used alone, reasonable combinations of these functions may be more helpful to users. For example, the combination of the functions *fixbond*, *addHs* and *setpchg* can be used to add the missing information of a molecule, which is necessary for the calculation of the energy.

(2). **PDBENT and TRIPOS**: functions in these two directories are primarily used to handle the files in PDB [12] and MOL2 [13] formats. The most important functions are *read_pdb*/*read_mol2* and *write_pdb*/*write_mol2*, which act as the controller of the data input/output stream in MORT. Each of these functions is composed of several sections, and in each section there is one function to parse the corresponding information. For example, *read_pdb* is used to get the molecule from a PDB formatted file. While parsing the file, it can recognize the first four letters of each line as its identity to determine which section it belongs to, and then the corresponding functions will be invoked to get the information from this line. Take "ATOM" for example: when "ATOM" has been recognized, the function *read_atom* will be used to parse the atom’s information (including its name, coordinate and type). And then an atom object will be created to store all the information.

(3). **SMARTS**: functions in this directory can handle two kinds of information: SMILES and SMARTS [14]. SMILES is the acronym of Simplified Molecular Input Line Entry Specification that has been widely used as a general-purpose chemical nomenclature and data exchange format, and SMARTS is the straightforward extensions of SMILES. The SMILES/SMARTS of a molecule is stored as a character string, which is the input parameter of the function *read_smiles*/*read_smarts*. These functions can extract the topology information of a molecule from the input string, and then store them into the database as a molecule. The whole process is not very complicated, and it will handle one character in each iteration. First, it will judge if the characters are in bracket "[]" or not, because the characters in or out of a bracket have different meanings. For example, "C" in "xxCxx" means that it is a normal carbon atom, but "C" in "[xxCxx]" may represent a carbon isotope. After the judgment, the corresponding functions will be used to analyze the character: *parse_charge* can be used to get the charge information from "+" and "-"; *parse_ring* can be used to recognize the digit outside a bracket as a starting/ending point of a ring; *parse_weight* can be used to parse the element’s weight that is listed in a bracket; *parse_alpha* can be used to interpret the element’s type, etc. Finally, all the information will be recorded into a molecule.

(4). **CAPBOX:** functions in this directory are mainly designed to add the solvent environment to an object. Four functions were designed to build different kinds of solvation environments: *solvatecap* can be used to add a solvate cap around the specified position of a solute; *solvatebox* can be used to add a solvate box to the solute in a cuboid way; *solvateoct* can be used to add the solvate box to a solute in a truncated octahedron way, which can reduce the number of the added solvent molecules; *solvateshl* can be used to add a solvate shell around the whole solute. The algorithms of these four functions are very similar. The process of *solvateshl* is illustrated in Figure 5 as an example.

Apart from the functions mentioned above, the function *addions* can be used to add positive/negative ions to the whole molecule.

(5). **ATMASK:** sometimes, users may want to get the selected partition of a molecule, such as the atoms within 5Å from the 4^th^ atom of a molecule. To achieve this goal, the function *mask_atom* was designed. This function can be employed to the specified atoms and residues. The argument of this function is a little complicated, and it is composed of some figures and symbols. For example, ":1-10" means to get the residues 1 to 10 from a molecule; "@4 < @5" means to return all the atoms within 5Å from the atom 4.

Following is the steps to interpret "@4 < @5". The first "@" indicates that this is something about atoms ("@" represents atoms and ":" stands for residues). The functions of class *atom_node* will then be invoked, and the following letters or numbers will be parsed as a MO in a specified molecule (here "4" represents the 4^th^ atom). The symbol "<" means to get the atoms/residues in a certain distance, and then it will invoke the function *parse* of class *dist_node* to interpret the next section. The previous MO will be regarded as a core. The second "@" indicates that the queried objects are atoms, and "5" equals to the threshold of the distance. Finally, the function *match* checks each atom in the molecule that satisfies the condition, and returns the qualified ones.

(6). **FORMAT:** functions defined here can be used to handle different kinds of formatted files. For example, the function *read_sdf* can be used to parse the molecule from a MOL/SDF formatted file, and the function *load_mdb* can be used to load the molecules from a database file, which contains a lot of molecules with the MOL2, MOL/SDF or OFF format. Once a molecule has been loaded, it will be stored in a database (represented by class *database_t*) with its name as the identity.

#### Property-related functions

The functions in this category can add, modify, save and delete different properties of a molecule, and all these functions are distributed in their corresponding directories.

(1). **ENEFRC:** functions in this directory are energy-related. The function *eval_bond*, *eval_angl* or *eval_tors* can be used to calculate the energy of bonds, angles or torsions. The non-bond energy can be calculated in two ways. The function *get_dir* can be used to calculate the energy based on periodic boundary condition (PBC) by using Ewald summation [15,16]. And the other function *nonbond_egb* can be used to calculate the polar contribution of desolvation for a non-periodic model by using the Generalized Born (GB) model [16] based on the following equations:

(1) $$E_{\text{GB}}=-\text{INVCHG}2*\left(1-\frac{1}{\varepsilon }\right)\sum_{j=i+1}^{n}\frac{q_{i}*q_{j}}{f_{\text{GB}}}$$

(2) $$\begin{array}{ll} \;\text{INVCHG}2 & =K_{e}*C^{2}*\mathrm{NA}/\left(10^{-10}*4.184\right)\\ & =332.05\text{Kcal}/\text{mol} \end{array}$$

(3) $$f_{\text{GB}}={\left(r_{\text{ij}}^{2}+\alpha_{\text{ij}}^{2}*\exp\left(-r_{\text{ij}}^{2}/{\left(2\alpha_{\text{ij}}\right)}^{2}\right)\right)}^{0.5}$$

(4) $$\alpha_{\text{ij}}^{2}=\alpha_{i}*\alpha_{j}$$

where *K*_e,_*C* and *NA* are electrostatic constant, Coulomb constant and Avogadro’s contant, respectively, *ε* is the dielectric constant of water (78.5), *INVCHG2* is a constant value of 332.05 kcal/mol, *r*_*ij*_ is the distance between two atoms, *α*_*i*_ and *α*_*j*_ are equal to the Born radii of atoms *i* and *j*. More detailed descriptions of the energy-related functions can be found in Additional file 1.

(2). **AMBFMT:** In this part of MORT, some functions were designed to read the AMBER and AMOEBA force fields and save the properties of a molecule into topology files. For example, *write_amber_prmtop* can be used to generate two files: topology (such as charge, bond, angle, etc.) and coordinate files (space information), which can be used as the input files for MD simulations. *read_frc/*read_amoeba_frc can be used to get the AMBER/AMOEBA force field parameters from an AMBER/AMOEBA parameter file and then store them into molecules.

For the better use of MORT, several commands have been defined in directory "plugin", which makes it very convenient to develop one serviceable application with just a few or sometimes just one command for the developers.

(3). **PLUGIN:** commands in this directory can be regarded as the MORT’s interface, and users can call these commands to accomplish many kinds of tasks with less effort than using functions. They can help the developers do more work in less time.

In each command class, apart from its constructor and destructor, one function is necessary:

virtual bool exec();

This function is a virtual function inherited from class *command_i*, which is the base class of all the command classes. *exec* performs variously in *command_i*’s child classes, reflecting the polymorphism of C++ language. Once one command class is declared, *exec* should be used to execute the corresponding commands at the end.

A lot of commands have be defined, for example, *source* can be used to interpret the files displayed in directory "dat/cmd", which contains the commands used for loading the force field parameters for the preparation of other operations (so *source* is usually the first thing that users need to do before executing any command); *merge* can be used to put the objects listed in its arguments together; *solvate* can be used to add the solvents around a molecule; *moloper* can be used to add the missing information to a molecule (mentioned in directory OBJFUN part), etc. These commands are all defined in their own classes, and users need to declare the class first before doing any operation. The details of the important functions and classes in MORT and the related information of important commands can be found in Additional file 1.

## Results and discussion

MORT can be used as a foundational library to develop new programs or software packages for computational biology and CADD. Usually, based on MORT, only a few codes are needed to solve a problem. For example, if users want to calculate the energy of a protein, the energy-related functions can be used conveniently. If the information of a molecule is incomplete, *fixbond*, *addHs* and other functions can work together to add the missing information.

**Figure 6:**
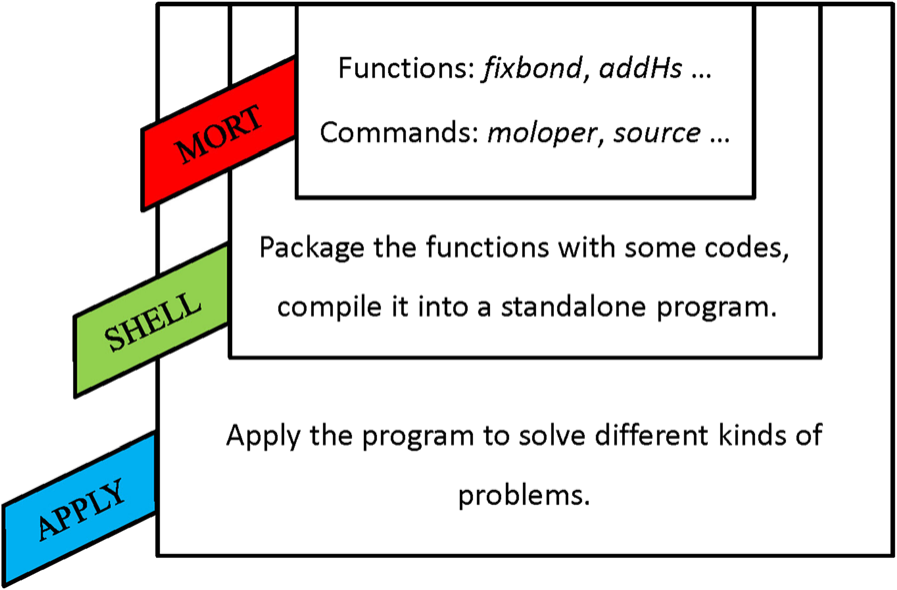
How to build a program based on MORT.

MORT can serve as the core of one program, what users need to do is to package this core with some necessary codes. They can be compiled as a standalone program to solve different kinds of problems. Figure 6 shows how a program can be builded based on MORT. The source code is provided with this manuscript as Additional file 2, and its installation script is documented in README. Some emamples are listed in *test* directory.

## Conclusions

**Figure 7:**
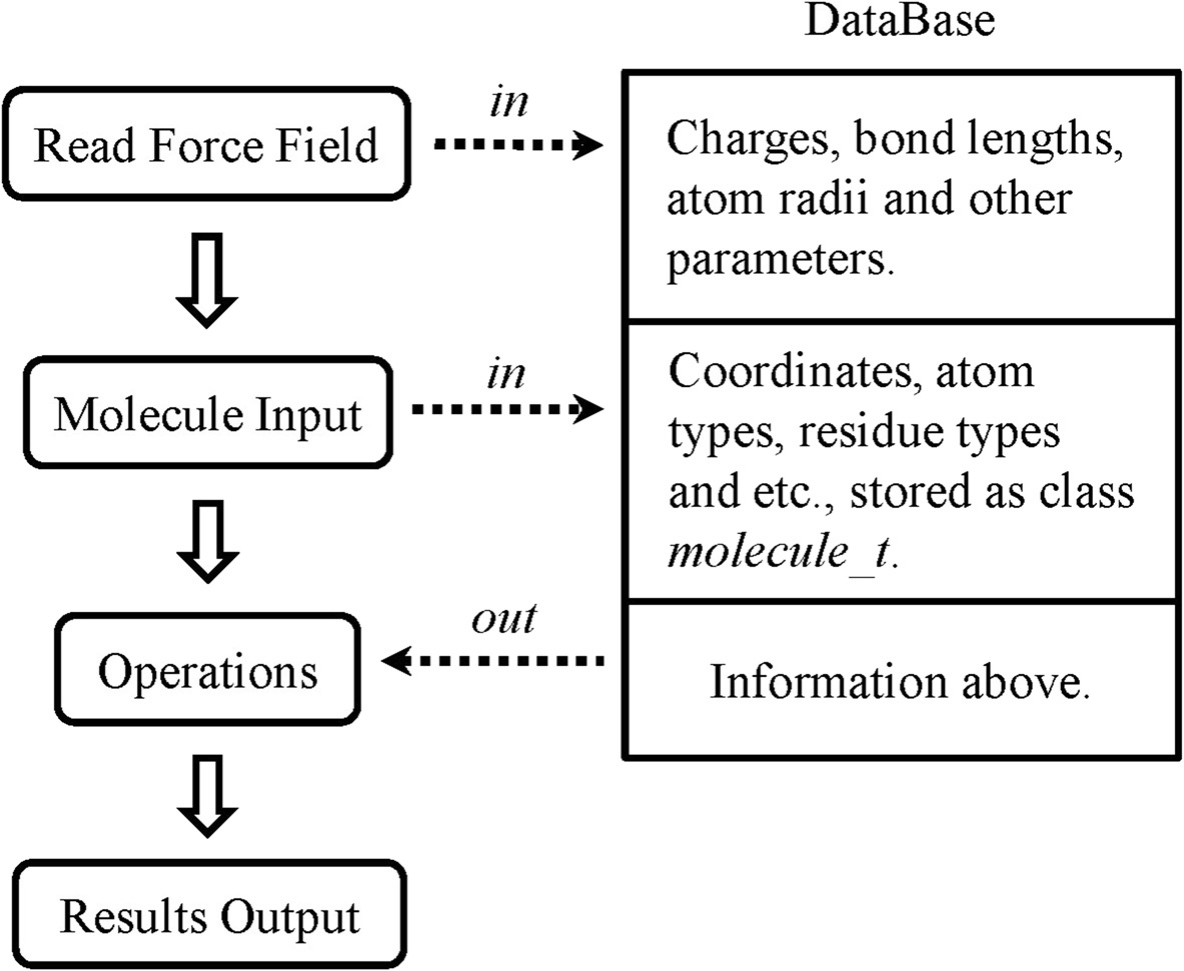
The working flowchart of MORT when dealing with some operations.

A C++ based library MORT is developed as a new foundation library for computational biology and CADD. This new library has many advantages, especially for its data structure and powerful functions. By employing the relational model instead of the hierarchical model to store data, less time is taken while iterating on the atoms and it solves the annoying problems such as determining the belongings of inter-residue bonds. In this model, all bonds and atoms are independent and extra entities are created to store the connection information and other relations between these entities. A lot of functions have been developed in this library, and Figure 7 shows how MORT works while dealing with some operations.

Moreover, many commands in AmberTools have been integrated into MORT. For example, *setpchg* can be used to call the standalone program in Antechamber [17] to assign atomic partial charges and *parmchk* can be used to call Parmchk to add missing force field parameters. With all these commands implemented, it is easy to create the topology file for a protein-inhibitor complex inside MORT without calling any outside programs.

## Availability and requirements

MORT is available at http://cadd.suda.edu.cn/MORT/index.htm, and it can be compiled into a static library on Linux platform. It’s written in C++ and the boost library (version 1.46.1 or newer) is needed.

## Competing interests

The authors declare that they have no competing interests.

## Authors’ contributions

TH, QZ and WZ instigated the project, designed and implemented the algorithms and developed the MORT library. TH, QZ, YL, JW and JZ drafted the manuscript. All authors read and approved the final manuscript.

## Supplementary Material

Additional file 1 The code organization, naming rules, and the detailed descriptions of the important classes and functions of MORT. Click here for file

Additional file 2 Source code of MORT. Click here for file
