# Supplementary material for: MORT: a powerful foundational library for computational biology and CADD
Source: J Cheminform. 2014 Jun 27;6:36. doi: 10.1186/1758-2946-6-36 (PMC4085231; doi:10.1186/1758-2946-6-36)
Supplement: Additional file 1 — The code organization, naming rules, and the detailed descriptions of the important classes and functions of MORT. [file 1758-2946-6-36-S1.doc]

# Supporting Information

MORT is a foundational library which is used for CADD and computational biology. It is written in C++, and employs the relational model to represent one molecule. A lot of functions have been developed in this library, and the most used functions are introduced below in details.

# CODE ORGANIZATION

The source code of mort is divided into 10 parts, and they are arranged in different subdirectories.

**AMBFMT:** Functions used to interpret the Amber’s file format.

**ATMASK:** Functions to get the selected partition of a molecule.

**CAPBOX:** Functions used to add water/ions to a molecule.

**COMMON:** All the basic operations are included here, such as constant definition, string algorithms and linear algebra algorithm.

**ENEFRC:** Functions used to calculate the energy.

**FORMAT:** Functions used to parse all kinds of molecule formats.

**OBJECT:** It contains the basic types of MORT.

**OBJFUN:** Functions used to operate the objects.

**PDBENT:** Functions used to interpret the PDB/ENT format.

**SMARTS:** Functions used to interpret the SMILES/SMART language.

**TRIPOS:** Functions used to interpret the Tripos mol2 format.

**PLUGINS:** Some useful commands defined in MORT.

# NAMING RULES

Class with “_t” represents that it an object, such as molecule_t, atom_t, bond_t, etc.

Class with “m_” represents that it stores the information of a molecule, such as m_adjacencys and m_components.

Class with “_ptr” represents that it is the pointer of an object, such as molecule_ptr (it is defined: typedef shared_ptr<molecule_t> molecule_ptr) and database_ptr.

Class with “_command” represents that it is the commands defined based on the functions, such as loadpdb_command and addions_command.

# CLASS DESCRIPTION

The most important classes of MORT are classes molecule_t and morf_t. molecule_t is used for storing a molecule and its connections, and it contains two member variables: m_components and m_adjacencys. m_components is used to store the objects, and m_adjacencys is used to store the relations between objects. morf_t is used to delete, modify and create the objects, and it is the father class of atom_t, bond_t, angl_t, dihe_t, impr_t and resd_t. Each of these classes represents one object, and almost all the operations are focused on these classes. Figure 1 shows one example to explain how a molecule is stored in MORT. CH4 is composed of 5 atoms and 4 bonds. Atoms are stored in the object ATOM, bonds are represented by the object BOND, and they are both included in m_components. The relations between atom and atom, atom and bond, bond and atom, and some others are stored in m_adjacencys.

Two methods can be used to iterate on objects by using MOITER (molecular object iterator, represent by *class iter_T*) or MORANGE (molecular object range, represented by *class range_T*).

MOITER works just like a pointer to *class morf_t*. The following member functions of *class molecule_t* return the starting and ending iterators of a certain type of MO:

*iter_t xxxx_begin ();*

*iter_t xxxx_end();*

Here, xxxx could be any of the eight 4 character ID of a molecular object type.

Class morange_t has the following member functions implemented:

*morf_t operator[](int id) const;*

*morf_t at(int id) const;*

MORANGE in a sense works just like an array of MOs. Class molecule_t has the functions “xxxxs()” that return the MORANGE of a certain type of MOs.

For getting and setting the information of objects, some functions are defined to accomplish these operations:

*void set_x( const string& pname, const value_type& v );*

*value_type get_x( const string& pname ) const;*

*bool get_x( const string& pname, value_type& v );*

The character ‘x’ in the names of these functions can be any of the following five characters: ‘i’ (for integer), ‘d’ (for double precision), ‘s’ (for string), ‘v’ (for numeric vector) and ‘a’ (for any other data type), while the value_type can be int, double, string, numvec, boost::any depending on the ‘x’. In these functions a property is retrieved by its name (of type string), which can be sometimes slow since it involves string comparison. So another type of these function are defined to accelerate the process by using hash ID (the detailed information can be found in the next part).

*void set_x( const long long& pid, const value_type& v );*

*value_type get_x( const long long& pid ) const;*

*bool get_x( const long long& pid, value_type& v );*

For the better use of molecules, class database_t is created to store the information of a molecule. It contains a vector of pair<string, entity_ptr>. Following are the member functions of database_t:

*bool has( const string& name ) const;*

*entity_ptr get( const string& name ) const;*

*molecule_ptr get_mol(const string& name) const;*

*database_ptr get_mdb(const string& name) const;*

*atmvec_ptr get_avec(const string& name) const;*

*bndvec_ptr get_bvec(const string& name) const;*

*namemap_ptr get_nmap(const string& name) const;*

*void set(const string& name, const entity_ptr& pmol );*

*void add(const string& name, const entity_ptr& pmol );*

*bool remove(const string& name);*

*iterator begin();*

*iterator end();*

*int size() const;*

entity_ptr is the shared pointer of root_t (the root class of molecule_t and database_t). The function get() can get the pointer of the entity with the name “name”, and the function set() can add one entity into database. The function add() works like set(), except that it does not check whether the entity with given name exists in the set or not.

A static database is defined in MORT, which is used to store the information of the molecule parsed from a PDB/ENT, MOL2, SDF/MOL, OFF or force field file.


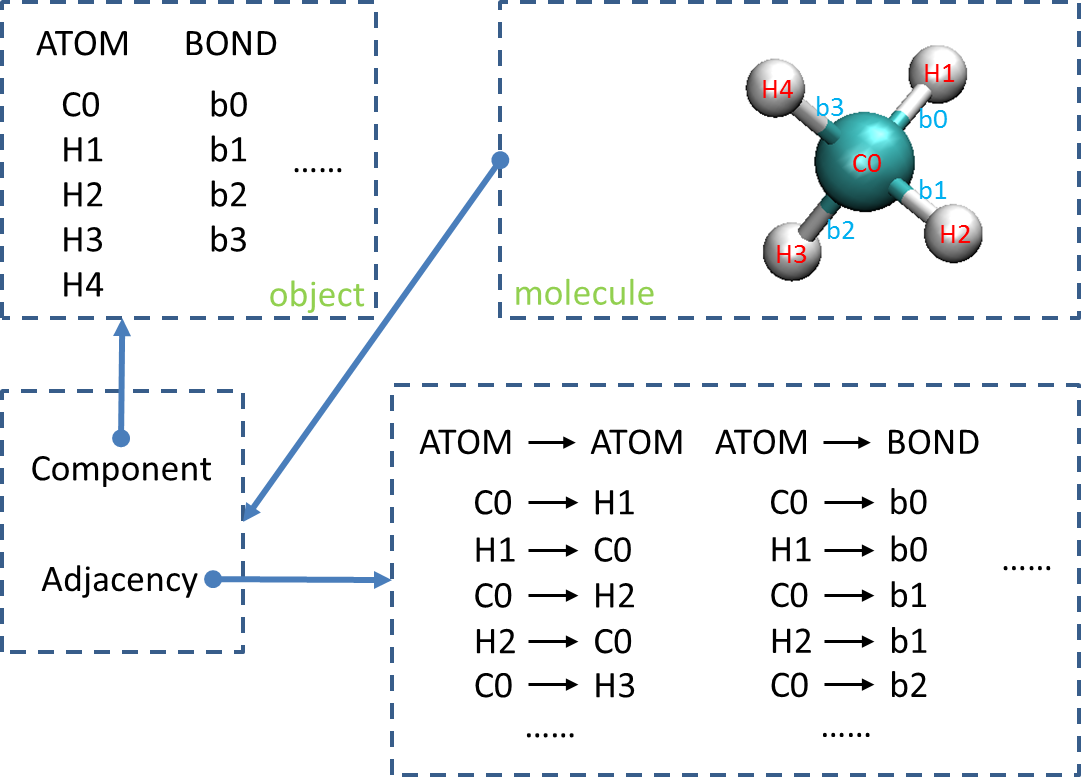


Figure 1: How CH4 is stored in MORT

# HASH DEFINITION

A lot of hash codes have been defined in common/hashcode.hpp to replace the string type in order to make this library faster, and some representative examples are shown below. Each hash is 10 letters long and only composed of letters, digital characters and underlines. More definitions can be seen in the source code, and “long long” is defined as hashid_t.

Some examples of the hash values are listed in Table 1, and they are generated by using their lowercase names:

Table 1: Some examples of the hash values

| **HASH IDs** | **HASH VALUES** |
| --- | --- |
| AAPOS | 47000000LLU |
| ADDITION | 2188132356920LLU |
| ALLIPHAITIC | 23194501875730040LLU |
| ALONE | 11094840LLU |
| AND | 5320LLU |
| ANGL | 714120LLU |
| AROM | 791080LLU |
| AROMATIC | 362394391080LLU |
| ATOM | 791160LLU |
| BOND | 213361LLU |
| CHAIN | 33792282LLU |
| …… | |

# FUNCTION DESCRIPTION

Functions are described in the order of the subdirectories in which they are located. Each subdirectory plays an important role in this library.

- **AMBFMT:** Amber is a very famous molecular simulation package developed by a lot of brilliant researchers. The Amber force fields have been widely used by other molecule simulation software packages for molecular simulation. In this part of MORT, some functions are designed in order to make it more convenient for developers who may what to parse the Amber force fields and the Amoeba force field. Some important functions are described here:
- **Read_frc**

Read force field paramters from an amber's paramter file, the paramters are stored in one molecule.

**Function:** void read_frc( std::istream& is, molecule_t& fp );

**Arguments:**

is: input stream.

fp: force field parameters.

- **Read_off**

Read object’s parameters from off file, off file contain water information which will be needed when adding solvation to the molecule. Different water models are in different off files, details can be seen in **CAPBOX** part.

**Function:** bool read_off( std::istream& is, molecule_t& m );

**Arguments:**

is:input stream.

m:parameter, which is stored in a molecule.

- **Read_amoeba_frc**

Read amoeba force field parameter. Amoeba Force Field is a useful Force Field, and is included in TINKER, which is a famous molecular modeling package.

**Function:** void read_amoeba_frc( std::istream& is, molecule_t& afp, molecule_t& bfp );

**Arguments:**

is: input stream.

afp:atomic part of force field parameters.

bfp:dipole part of force field parameters.

- **Write_amber_prmtop**

Save the parameters of molecule into topology file. This is a very useful function, topology file contains a lot descriptions of molecule, such as atom, bond, angle and so on. All these can be used by other programs for further analysis.

**Fucntion:** void write_amber_prmtop( ostream& os, molecule_t& m, const molecule_t& ffp );

**Arguments:**

os: output stream.

m: molecular object.

ffp: force field parameter.

- **ATMASK:** sometimes, users may want to get the copy of the part of the molecule, to this end, functions here can be uesd to accomplish this intention.
- **mask_atom**

get the specified part of one moelcule, and return the atoms or residues as a new moelcule in MORT.

**Function:** atomvec_t mask_atom( const molecule_t& mol, const string& mask );

**Arguments:**

mol: molecular object.

mask: the expression to specify the partition of the moelcule. It can start with “:” or “@”, which means to select by residues or atoms. If it starts with “:”, then the following will have two selections: “:{numlist}” (such as “:1-17”) and “:{namelist}” (“:GLY,ARG”). If “@”, the type can be three: “@{numlist}” (@1-17), “{namelist}” (“@CA, O, H”) and “@%{typelist}” (@%N3,CT). There also exist some operators: ("<:", ">:", "<@", ">@"), "!", "&" and "|" (listed in the order of descending priority). Users can use operators to make the expression more sepcific. For example, “:4 <@7” means to get all the atoms within 7 Å of residue 4; “:ARG, GLY & @CA,O” means to return the CA and O atom of residue “ARG” and “GLY”.

- **CAPBOX:** Four solvation functions were designed to add solvent environment. Apart from adding solvent box, the functions that can add ions to solute are defined in this part as well. Brief introduction of these functions can be found below:
- **Solvatecap, Solvatebox, Solvateoct, Solvateshl**

*Solvatecap* is used to add a solvate cap around the solute’s specified position, *solvatebox* can add solvate box to one molecule in a cuboid way, *solvateoct* is used to add solvate box to one molecule in a truncated octahedron way, and *solvateshl* can add a solvate cap around the solute. If these functions are used, “#include <capbox/solvate.hpp>” should be in the head of the source code.

**Function:** void solvatecap(molecule_t& mol, const molecule_t& svt, const numvec& capcnt, double caprad, double closeness);

**Function:** void solvatebox(molecule_t& mol, const molecule_t& svt, double buffer, double closeness, bool iso);

**Function:** void solvateoct( molecule_t& mol, const molecule_t& svt, double buffer, double closeness );

**Function:** void solvateshl( molecule_t& mol, const molecule_t& svt, double shlext, double closeness );

**Arguments:**

mol: molecule mol (solute).

svt: molecule svt (solvent).

capcnt: center of cap.

caprad: radius of cap.

buffer: the buffer between solute and solvate.

closeness: the closeness between solute and solvate.

iso: a boolean to judge if the box is a cube.

shlext: extent of the shell.

- **Addions**

Add ions to one molecule, there exists another version of this function which can add two kinds of ions to one molecule with two more arguments: “const molecule_t& ion2” and “int numion2”, ion1 and ion2 should carry the opposite charge.

**Function:** void addions( molecule_t& m, const molecule_t& ion, int nion, double shlext, double res );

**Arguments:**

m: molecular object.

ion: ions.

nion: number of ions.

shlext: the thickness of the shell.

res: resolution of the whole system.

- **COMMON:** All the basic operations are included in this directory, such as the constant definition, string algorithms and linear algebra algorithm. These functions will be used frequently, and are very necessary for many others functions. Constant definition has been introduced in the **HASH DEFINITION** part. The functions used to do linear transformation to a structure are included in this part, such as rotate, transform and so on. The functions used to deal with the features of plane and stereo can also be found here.
- **Transform**

Transform the molecule according to the vector. The second arguments should have 16 elements, it can be regarded as a 4*4 matrix. The top-left 3*3 is a transform matrix, and the last element can be regard as the scale.

**Function:** void transform ( vector<double>& crd, const double* mat );

**Arguments:**

crd: coordinate of the molecule.

mat: the pointer of transformation matrix.

- **Rotate**

Rotate the molecule around rotation axis. The rotation method is called “Rodrigues rotating function”. Target vector rotates around the axis, *sita* is the rotation angle and the direction is clockwise.

**Function:** numvec& rotate( numvec& vec, const numvec& axs, double ang );

**Arguments:**

vec: coordinate of the molecule.

axs: rotation axis.

ang: rotation angle.

- **Translate**

Translate the vector according to the translation vector.

**Function:** void translate ( vector<double>& crd, const numvec& sft );

**Arguments:**

crd: coordinate of the molecule.

sft: translation vector.

- **Dist, Angl, Tors**

*Dist* can calculate the bond length between two atoms, *Angl* will return the angle composed by three atoms and *Tors* is used to calculate the torsion angle composed by 4 atoms.

**Function:** double dist( const numvec& v1, const numvec& v2 );

**Function:** double angl( const numvec& v1, const numvec& v2, const numvec& v3 );

**Function:** double tors( const numvec& v1, const numvec& v2, const numvec& v3, const numvec& v4 );

**Arguments:**

v1, v2, v3 and v4: coordinates of atoms.

- **Zmatrix**

This function is used to define the Cartesian coordinates of ATOMs within object using internal coordinates. It is composed by 3 separate functions (see below), the first function is used to transform second atom’s internal coordinate into cartesian coordinate, the second function is for the third atom, and the last function is used for the following atoms.

**Function:** numvec zmatrix(const numvec& p1, double dist);

**Function:** numvec zmatrix(const numvec& p1, double dist, const numvec& p2, double angl);

**Function:** numvec zmatrix(const numvec& p1, double dist, const numvec& p2, double angl, const numvec& p3, double tors);

**Argument:**

p1, p2, p3: cartesian coordinates of the atoms

dist: distance between two atoms

angl: angles generated by three atoms

tors: torsions composed by four atoms

- **Plane**

Return the plane composed by 3 points, the result is a 4-element vector (n1, n2, n3, d), and the plane is determined by the following equation: n1*x+n2*y+n3*z+d=0.

**Function:** numvec plane( const numvec& v1, const numvec& v2, const numvec& v3 );

**Arguments:**

v1, v2 and v3: coordinates of 3 points.

- **ENEFRC:** Energy can be divided into two parts: bond-related energy and non-bond energy. Functions used to calculate the energies of bond, angle, torsion and out-of-plane are included in the previous one. And the electrostatic potential, van der Waals potential and energy based on the Generalized Born (GB) model can be calculated by the non-bond energy functions. Formulas’ definitions are listed in Table 2.
- **Eval_bond, Eval_angl, Eval_tors, Eval_oops**

These four functions are used to calculate the bond related energy, their functions are self-explanatory.

**Function:** double eval_bond( const molecule_t& mol );

**Function:** double eval_angl( const molecule_t& mol );

**Function:** double eval_tors( const molecule_t& mol );

**Function:** double eval_oops( const molecule_t& mol );

- **Nonbond_egb**

This function is used to calculate non-bond energy including electrostatic potential, Van Der Waal potential and energy based on GB model, these energy are included in a numvec.

**Function:** numvec nonbond_egb(const molecule_t& m, const ctrlparm_t& p);

**Arguments:**

m: molecule m.

p: parameters for the calculation of energy. It contains several flags: igb, cut, rgbmax and offset. They can be separated by using “ ”, “,” and “:”. “rgbmax” is set to be 25.0 and “offset” is set to be 0.09 by default. “igb” is used to determine if periodic boundary conditions (igb=0) should be used, and “cut” is the cutoff of non-bond interaction.

- **Get_dir**

This function is used to calculate non-bond energy in the periodic boundary conditions by using the Ewald summation.

**Function:** numvec get_dir(const molecule_t& m, const ctrlparm_t& p);

**Arguments:**

m: molecule m.

p: parameters for the calculation of energy. Details can be seen in nonbond_egb section.

Table 2: The energy definition in MORT

| **Bonding Energy** | **Equation** |
| --- | --- |
| bond potential | K_b_*(*l* - *l*_0_)^2^ |
| angle potential | K_a_*(*θ* - *θ*_0_)^2^ |
| torsion potential | K_t_*[1 + cos(n*t*+*t*_0_)] |
| improper torsion potential | K_i_*[1 + cos(n*i*+*i*_0_)] |
| **Nonbond Energy** |  |
| electrostatic potential | INVCHG2*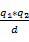 |
| Van Der Waal potential | 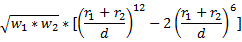 |
| potential on GB model (polar solvation) | - INVCHG2*(1 - 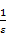)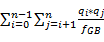 |
| potential on PBC | A little complicated, not shown here |

In Table 2, K_b_, K_a_, K_t_ and K_i_ represent their corresponding force constants, and *l*_0_, *θ*_0_, *t*_0_ and *i*_0_ are their equilibrium constant. *q*_1_ and *q*_2_ are the charges of two atoms, *r*_1_ and *r*_2_ equal to the van der Waal radius, *w*1 and *w*2 are the depth of potential well, and *d* is the distance between two atoms. INVCHG2 is a constant of 332.05kcal/mol, and it is calculated by using the following equation:


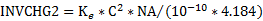
 = 332.05 kcal/mol (1)

For the polar solvation energy,
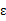
 is the dielectric constant of water (78.5), and *f*_GB_ is defined as follows:


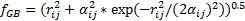
 (2)


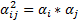
 (3)

Where
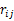
 is the distance between two atoms, and
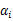
 and
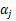
equal to the Born radii of atoms *i* and *j*.

- **FORMAT:** The functions in this part are used to parse different kinds of molecule formats, including the MDL SDF format, the PDB format and the TRIPOS MOL2 format. There are also some functions that can be used to load or save the database, and the database represents the set of molecules or parameters.
- **Read_sdf**

Read the molecule from SDF format input stream.

**Function:** void read_sdf( istream& is, molecule_t& mol );

**Arguments:**

is: input stream.

mol: molecule mol.

- **Write_sdf**

Save the molecule in stream of SDF format.

**Function:** void write_sdf( ostream& os, const molecule_t& mol );

**Arguments:**

os: output stream.

mol: molecule mol.

- **Load_mdb**

Load database from input stream according to format code.

**Function:** void load_mdb( std::istream& is, database_t& db, const hashid_t& format );

**Arguments:**

is: input stream.

db: database db.

format: predefined format code, can be SDF, MOL2 and OFF.

- **Save_mdb**

Save database to stream according to format code. This function can be used to save a set of molecules into one database file.

**Function:** void save_mdb( std::ostream& os, database_t& db, const hashid_t& format );

**Arguments:**

os: output stream.

db: database db.

format: predefined format code, can be SDF, MOL2 and OFF.

- **OBJECT:** Object class atom_t, bond_t, angl_t, dihe_t, impr_t and resd_t are designed here. All these class are extended from morf_t, and they have some common basic functions defined in this part. Class mole_t can store one molecule’s information, and the details can be seen in **CLASS DESCRIPTION**.

Common functions for object class: *create*, *get* and *has*. *Create* is used to create one object, *get* can return the object that is composed by the given elements, and *has* is used to check if there is one object composed by the given elements.

For example: in angl.hpp

**Function:** static angl_t create( atom_t& a1, atom_t& a2, atom_t& a3 );

**Function:** static angl_t get( const atom_t& a1, const atom_t& a2, const atom_t& a3 );

**Function:** static bool has( const atom_t& a1, const atom_t& a2, const atom_t& a3 );

- **OBJFUN:** The functions in OBJECT are very basic, and more operations dealing with objects can be found in this directory. Too many functions have been developed here, and they operate on different objects. For example, to ATOM, *get_atom_hybrid* is used to get one atom’s hybridization, *get_atom_valence* returns the valance of a specified atom, *get_atom_pie* will return the atom’s contribution to the aromatic ring; To BOND, *bond_bydis* can create the bond between two atoms when the distance between them is smaller than the given cut-off, *create_tor2* will return the object torsion generated by 4 atoms; and for the whole molecule, some functions can be seen below:
- **Fixbond**

This is a very useful function, it adopts several rules to set the bond order of molecule m. It can be used to add bonds’ information to the molecule file, such as PDB formatted file, which doesn’t contain bonds’ information of proteins.

**Function:** void fixbond( molecule_t& m );

**Arguments:**

m: molecule m.

- **AddHs**

Add hydrogen to molecule, the molecule should have correct bond order assigned.

**Function:** void ( molecule_t& mol );

**Arguments:**

mol: molecule mol.

- **Merge**

Merge two molecules into one, return the newly created residue.

**Function:** resd_t merge( molecule_t& m, const molecule_t& s, int rid=-1 );

**Arguments:**

m and s: molecule m and s.

rid: the id of residue in molecule m.

- **PDBENT:** PDB (Protein Data Bank) is a repository for the 3-D structural data of large biological molecules, such as proteins and nucleic acids. A PDB file is composed of several parts, including ATOM, CONNECT, HYDBND and etc., and detailed information is available at <http://www.wwpdb.org/docs.html>. The functions that can parse the PDB format are listed in this directory.
- **Read_pdb**

Read the molecule from the PDB file.

**Function:** bool read_pdb( istream& is, molecule_t& mol );

**Arguments:**

is: input stream.

mol: molecule mol.

- **Write_pdb**

Write the molecule mol into PDB formatted file.

**Function:** bool write_pdb( ostream& os, const molecule_t& mol );

**Arguments:**

os: output stream.

mol: molecule mol.

- **SMARTS:** SMARTS/SMILES is a language designed for describing molecule patterns. SMARTS/SMILES is just a string, and the topology information of a molecule is stored in this string. The functions used to parse this formatted string are developed.
- **Read_smiles**

Load the molecule from SIMLES string.

**Function:** bool read_smiles( const char* exp, molecule_t& mol );

**Arguments:**

exp: SIMLES expression, a string.

mol: molecule mol.

- **Read_smarts**

Load the molecule from SMARTS string.

**Function:** bool read_smarts( const char* exp, molecule_t& mol );

**Arguments:**

exp: SMARTS expression, a string.

mol: molecule mol.

- **TRIPOS:** Except from SDF and PDB, the MOL2 format is also popular. A MOL2 file is a complete, portable representation of a SYBYL molecule, it contains all the information needed to reconstruct one molecule. It is also constitutive of several sections, such as @<TRIPOS>ATOM, @<TRIPOS>BOND, and so on.
- **Read_mol2**

Read the molecule from mol2 formatted file.

**Function:** void ( istream& stream, molecule_t& mol );

**Arguments:**

stream: input stream.

mol: molecule mol.

- **Write_mol2**

Output the molecule’s information.

**Function:** void write_mol2( ostream& stream, const molecule_t& mol );

**Arguments:**

stream: output stream.

mol: molecule mol.

- **PLUGINS:** In order for the better use of mort, we developed lots of commands. And with all these commands, researchers will do less work if they use mort. There is one command named *source*, which is used to interpret the force filed files displayed in dat/cmd.

MORT has been tested at ubuntu 12.04 and Red Hat Enterprise Linux Server release 5.5 (Tikanga).

# Examples

1. The following codes can be used to calculate the energy of a molecule (PDB entry: 2PMV), and only a few lines are needed to accomplish this goal. The code is located in the test directory.

#include <iostream>

#include <plugin.hpp>

#include <object.hpp>

#include <objfun.hpp>

#include <format.hpp>

#include <ambfmt.hpp>

#include <enefrc.hpp>

using namespace std;

using namespace mort;

int main**()**

**{**

string file **=** "../dat/cmd/leaprc.ff99SB"**;** //load parameters from force field

ifstream is**(**file**.**c_str**());**

source**(**is**);**

loadpdb_command pdb**(**"loadpdb"**,**"2PMV"**,**"2PMV.pdb"**,**""**);**//load pdb

pdb**.**exec**();**

molecule_ptr mol**=** content**().**get_mol**(**"2PMV"**);** //get the molecule with its name

energy_command energy**(**"2PMV"**,**"igb,1:cut,12"**);** //calculate the energy according to the parameters

energy**.**exec**();**

**}**

2. Sometimes, the mirror structure of a molecule is needed, and the following code can be used to reflect one molecule according to the given plane (only PDB format is considered, and the operations for the other formats are same). A lot of commands have been created, and their information can be seen in Command’s Description.

#include <iostream>

#include <fstream>

#include <string>

#include <objfun.hpp>

#include <object.hpp>

#include <common.hpp>

#include <plugin.hpp>

using namespace std;

using namespace mort;

void reflect(numvec & plane,numvec & pos);

int main (int argc,char *argv[])

{

if(argc!=7)

{

cerr << "one plane should have 4 elements" << endl;

return 1;

}

ifstream input(argv[1]);

ofstream output(argv[2]);

if(!input)

{

cerr << "Input file not existS" << endl;

return 1;

}

string file = "dat/cmd/leaprc.ff99SB"; //load the parameters before load the molecule

ifstream is(file.c_str());

source(is);

loadpdb_command load("loadpdb", "2PMV", argv[1], "");

load.exec();

molecule_ptr mol= content().get_mol("2PMV"); //get the molecule

numvec plane(4); //generate the plane

int k = 3;

for(int i=0;i<4;i++) //set plane's value

{

plane[i] = atof(argv[k]);

k++;

}

atomiter_t iter = mol->atom_begin(); //the iterator of atoms

for(;iter!= mol->atom_end();iter++)

{

numvec pos= iter->get_v(POSITION); //get the position of each atom

reflect( plane, pos ); //reflection transformation of each atom

iter->set_v(POSITION, pos ); //set the new position

}

savepdb_command save("2PMV",argv[2]);

save.exec();

}

void reflect(numvec &p,numvec &v) //reflect one vector according to the plane

{

numvec r(3);

double pp = p[0]*p[0]+p[1]*p[1]+p[2]*p[2];

double p0 = p[0]*p[0]-p[1]*p[1]-p[2]*p[2];

double p1 = p[1]*p[1]-p[0]*p[0]-p[2]*p[2];

double p2 = p[2]*p[2]-p[0]*p[0]-p[1]*p[1];

r[0]=-2*p[0]*(v[1]*p[1]+v[2]*p[2]+p[3])-v[0]*p0;

v[0]=r[0]/pp;

r[1]=-2*p[1]*(v[0]*p[0]+v[2]*p[2]+p[3])-v[1]*p1;

v[1]=r[1]/pp;

r[2]=-2*p[2]*(v[0]*p[0]+v[1]*p[1]+p[3])-v[2]*p2;

v[2]=r[2]/pp;

}

# Additional functions:

Setpchg: Calls the standalone program in Antechamber to assign atomic partial charges.

Parmchk: Add missing force field parameters by invoking Parmchk.

These functions are contained in Amber, and please make sure that you have Amber (AmberTools at least) installed in your computer, and the environmental variable AMBERHOME is set correctly if you want to use these functions.
